# The Intestinal Spirochete *Brachyspira pilosicoli* Attaches to Cultured Caco-2 Cells and Induces Pathological Changes

**DOI:** 10.1371/journal.pone.0008352

**Published:** 2009-12-17

**Authors:** Ram Naresh, Yong Song, David J. Hampson

**Affiliations:** Animal Research Institute, Murdoch University, Murdoch, Australia; Charité-Universitätsmedizin Berlin, Germany

## Abstract

**Background:**

*Brachyspira pilosicoli* is an anaerobic spirochete that has received relatively little study, partly due to its specialized culture requirements and slow growth. This bacterium colonizes the large intestine of various species, including humans; typically, a dense layer of spirochete cells may be found intimately attached by one cell end to the surface of colonic enterocytes. Colonized individuals may develop colitis, but the mechanisms involved are not understood. The current study aimed to develop an *in vitro* model to investigate this process.

**Methodology/Principal Findings:**

Four strains of *B. pilosicoli* were incubated at a high multiplicity of infection with monolayers of a human colonic adenocarcinoma cell line (Caco-2 cells). One strain isolated from a pig (95/1000) and one from a human (WesB) attached to the monolayers. Colonization increased with time, with the Caco-2 cell junctions being the initial targets of attachment. By electron microscopy, individual spirochete cells could be seen to have one cell end invaginated into the Caco-2 cell membranes, with the rest of the spirochete draped over the Caco-2 cell surface. After 6 h incubation, the monolayer was covered with a layer of spirochetes. Colonized monolayers demonstrated a time-dependent series of changes: staining with labelled phalloidin identified accumulation of actin at the cell junctions; ZO-1 staining revealed a loss of Caco-2 tight junction integrity; and Hoechst staining showed condensation and fragmentation of nuclear material consistent with apoptosis. Using quantitative reverse transcription PCR, the colonized monolayers demonstrated a significant up-regulation of interleukin-1β (IL-1β) and IL-8 expression. *B. pilosicoli* sonicates caused significant up-regulation of IL-1β, TNF-α, and IL-6, but culture supernatants and non-pathogenic *Brachyspira innocens* did not alter cytokine expression.

**Conclusions/Significance:**

The changes induced in the Caco-2 cells provide evidence that *B. pilosicoli* has pathogenic potential, and give insights into the likely *in vivo* pathogenesis.

## Introduction

The intestinal spirochete *Brachyspira pilosicoli* colonizes the large intestine of a variety of species of animals and birds, as well as human beings [1]. Infection is common in intensively farmed chickens and pigs, in which the spirochete is considered to be an important enteric pathogen [2], [3]. In humans, infection is common in homosexual males and HIV patients in developed countries [4], [5], but also it occurs frequently in people in developing countries, especially those living in crowded and unhygienic conditions [6], [7]. Recently, large numbers of intestinal spirochetes have been found in stool samples from patients with cholera, and it has been suggested that they may exacerbate the disease [8].

A characteristic feature of colonization with *B. pilosicoli* is the intimate end-on or “polar” attachment of spirochete cells to the luminal surface of colonic and rectal epithelial cells, in a condition called “intestinal spirochetosis” or “colonic spirochetosis” [1]. This description was first made in colonic biopsy samples from humans where the associated dense layer of attached spirochetes was described as a “false brush border” [9]. Subsequently, a similar condition was described in pigs [10], and eventually it was shown that strains of the same spirochete species (now called *B. pilosicoli*) could cause the condition in both humans and pigs [11], [12]. Humans also may be colonized with the distinct species *Brachyspira aalborgi*, which similarly attaches to colonic enterocytes by one cell end [13], [14].

*Brachyspira pilosicoli* is difficult to isolate as it is anaerobic and grows slowly, and, despite its potential importance as a pathogen, it has not been extensively studied. Very little is known about virulence determinants in this spirochete, apart from the fact that it appears to lack the attachment and invasion determinants encoded by the *inv*, *ail* and *yadA* genes of *Yersinia enterocolitica*, the *eae* gene from enteropathogenic *Escherichia coli*, and a virulence plasmid of *Shigella flexneri* [15]. Progress has been hampered by a lack of genomic information for this spirochete, an absence of means for genetic manipulation, and a lack of *in vitro* models in which to study the pathogenesis of infection.

Pigs, chickens and mice have been used experimentally as models of *B. pilosicoli* infection, using spirochete strains isolated from various species, including humans [16]–[21]. In these models, as in the natural infections, one cell end of the spirochetes can be seen invaginating into the mature columnar cells without penetrating the host cell membrane. Often hundreds of individual spirochete cells can be seen attached to the surface of each enterocyte, forming a dense mat of spirochete cells overlaying the epithelium. In the only previous published study using *B. pilosicoli* to infect intestinal epithelial cell lines, a diffuse attachment of spirochetes was obtained, but the characteristic attachment by one cell end was not observed, and pathological changes similar to those that occur *in vivo* were not induced [22].

The aim of the current study was to establish an *in vitro* model to study the interactions of *B. pilosicoli* with enterocytes, and to gain insights into the pathogenesis of the infection that have not been documented previously.

## Materials and Methods

### Spirochete Strains and Growth

Two Australian strains of *Brachyspira pilosicoli* isolated from human beings (WesB and Karlton), and two from pigs (95/1000 and Cof-10), as well as the *Brachyspira innocens* type strain B256^T^, were obtained as frozen stock from the culture collection held at the Australian Reference Centre for Intestinal Spirochetes, School of Veterinary and Biomedical Sciences, Murdoch University. The cells were thawed and grown in Kunkle's pre-reduced anaerobic broth, containing 2% (v/v) fetal bovine serum and 1% (v/v) ethanolic cholesterol solution [23]. Broth cultures were incubated at 37°C on a rocking platform for 3–5 days, and spirochete growth was monitored daily by examining aliquots under a phase contrast microscope. Cell numbers were established by direct counting in a Neubauer counting chamber under a phase contrast microscope at a 400× magnification.

### Culture Supernatants and Sonicates

Culture supernatants were prepared by centrifuging 1 ml of broth culture containing actively motile mid-log phase spirochete cells (10^8^/mL) at 10,000 X *g* for 40 min, and carefully aspirating the supernatant. To prepare cell sonicates, duplicate broths were centrifuged at 5,000 X *g* for 15 min, and the pellet was resuspended in phosphate buffered saline (PBS; 3.2 mM Na_2_HPO_4_, 0.5 mM KH_2_PO_4_, 1.3 mM KCl, 135 mM NaCl, pH 7.4). This material was disrupted (Ultrasonic Processor XL, Misonix Incorporation, Farmingdale, NY) at 4°C with six bursts of 10 s each, centrifuged at 10,000 X *g* for 25 min, and the supernatant was aspirated and used as the source of sonicate.

### Cell Cultures

The intestinal epithelial cell line Caco-2, derived from a human colonic adenocarcinoma (HTB-37; ATCC, Manassas, VA), was grown in Dulbecco's modified Eagle medium (DMEM) (Sigma Chemical Co., St. Louis, MO), supplemented with 10% heat-inactivated fetal bovine serum, 1% L-glutamine, 100 U of penicillin/ml, 100 µg of streptomycin/ml, and 0.25 µg of amphotericin B/ml (all from Sigma). The cells were grown at 37°C in a humidified atmosphere containing 5% CO_2_. The culture medium was changed every 2–3 d, and when appropriate the cells were passaged with 2 X trypsin-EDTA (Sigma).

### Attachment Assays

For electron microscopy, the monolayers were grown in 48 well plates (Greiner Bio-One, Frickenhausen, Germany) with sterile 13 mm thermanox inserts (ProSciTech, Thuringowa, QLD, Australia) in the bottom of each well. The wells were seeded with trypsinized Caco-2 cells at a concentration of 4×10^4^ cells per well, and incubated at 37°C under 5% CO_2_ tension for 10–14 d. The growth of the monolayers was monitored and the DMEM was replaced as required. Well-grown, confluent and fully differentiated Caco-2 cells were used for the attachment assays. Actively motile cultures of the *B. pilosicoli* strains in mid-log phase were used in the assays. The spirochetes were harvested from the broth culture by centrifuging at 800 X *g* for 20 min, and then the pellet was resuspended in the DMEM. One ml of the respective suspensions containing 10^8^ spirochete cells was added per well, to give a multiplicity of infection of approximately 100. Control wells received 1 ml of DMEM. Incubation was for 2, 4 and 6 h, with 3 replicates for each spirochete strain at each time point. All assays were repeated at least three times. At the end of the incubation period the medium containing the bacteria was aspirated, the wells were filled with PBS, aspirated, and washed again three times to remove any remaining unattached bacteria, before they were processed for electron microscopy.

### Electron Microscopy

The cells on the washed inserts were fixed with 2.5% glutaraldehyde at 4°C overnight, and then were washed five times with 0.07 M Sorensen's buffer (3 parts 0.01 M Na_2_HPo_4_ and 1 part 0.01 M KH_2_Po_4_). The inserts were post-fixed in 1% aqueous osmium tetroxide at 4°C for 1 h, and washed three times with 70% ethanol before being dehydrated through an ethanol series. For scanning electron microscopy (SEM), the inserts were removed from the wells, critically point dried on a Balzers Union critical point dryer with carbon dioxide as the exchange medium, and mounted on stubs using double-sided adhesive tape. Stubs were sputter coated with gold to a thickness of 90 nm in a Balzers sputter coater, and examined using a Philips XL 20 scanning electron microscope. A semi-quantitative scoring system for the extent of attachment at the different time points was used. An operator blinded to the origin of the samples examined 12 fields at a 2,000× magnification, and scored each field from 0 to 5, where 0 indicated no attached spirochetes and 5 indicated that the surface of the whole field was covered with attached spirochetes.

For transmission electron microscopy (TEM), the dehydrated cells were processed for infiltration with propylene oxide (2 changes over 20 min), then with a propylene/resin mix (60/40) for 1 h at 4°C, and finally with absolute resin on a rotary mixer at 25°C overnight. The cells were embedded with pure resin at 60°C for 24 h, and 90 nm sections of the monolayers were cut with an ultra-microtome and mounted on carbon coated grids. The grids were stained with freshly prepared uranyl acetate and lead citrate and were examined using a Philips 1 CM -100 transmission electron microscope.

### Preparation of Caco-2 Monolayers for Staining

A 400 µl volume of trypsinized Caco-2 cells (10^6^ cells/ml) was added to each well of an 8-well Lab-Tek™ chamber slide system (Nalge Nunc International, Naperville, IL), and these were incubated at 37°C in a humidified atmosphere containing 5% CO_2_. The medium was changed every 24 h, and assays were conducted when the monolayers were confluent and fully differentiated. A total of 10^7^ cells of *B. pilosicoli* 95/1000 resuspended in 400 µl DMEM was added to the slides, and they were incubated for 2, 4 or 6 h. The assays were run in triplicate. Control slides were incubated for the same time with *B. pilosicoli* broth supernatant, sterile uninoculated broth, and DMEM. The slides were washed three times with PBS before processing.

### ZO-1 and Hoechst Fluorescent Staining

Staining for the tight junction protein zonula occludens-1 (ZO-1) with labelled antiserum, and for DNA using Hoechst staining, was performed on the washed Caco-2 monolayers. The cells were fixed and permeabilized for 20 min at 4°C by adding 400 µl of cold methanol to each well. For ZO-1 staining, after two washes with PBS 100 µl of primary antibody (rabbit anti-ZO1; Zymed Laboratories Inc., San Francisco, CA) diluted 1∶100 in PBS containing 2% fetal bovine serum was added to each well, and these were incubated at 37°C in a humid chamber for 1 h. The cells were washed twice with PBS, and 100 µl of secondary antibody (Alex 555-conjugated goat anti-rabbit; Invitrogen Pty. Ltd, Mount Waverley VIC, Australia) diluted 1∶2,000 in PBS containing 2% fetal bovine serum was added to each well. After incubating for 1 h at 37°C in a dark humid chamber, the cells were washed twice with PBS. For nuclear staining, 400 µl of a 1 µM Hoechst solution (Molecular Probes, Eugene, OR) was added to each well, and these were incubated in the dark for 5 min at 25°C. After two washes with PBS, the coverslips were mounted in the dark using aqua polymount (Polysciences, Inc., Warrington, PE). The slides were stored in the dark until they were examined under an Olympus BX51 epifluorescent microscope with Green Excitation Filter UMWG2 for ZO-1 and Ultra Violet Excitation Filter UMWU2 for nuclear staining. The relative numbers of condensed and non-condensed nuclei were counted in 6 visual fields at a 100× magnification, and the percentages for the different treatments were compared using Student's *t*-test.

### Staining of Filamentous Actin

The washed Caco-2 cells were fixed in 3% neutral buffered formalin for 20 min at 25°C. They were washed three times with PBS, and were made permeable by treating with 0.1% Triton X-100 in PBS for 5 min. After three washes in PBS, the cells were treated with a 5 µg/ml solution of fluorescein isothiocyanate-phalloidin (Sigma) in PBS for 20 min. The cells were washed three times with PBS and were mounted with glycerol-PBS (3∶1). The monolayers were examined under an Olympus BX51 epifluorescent microscope (FITC filter, U-MWIB2).

### Cytokine Expression Assays

Two experiments were conducted using quantitative reverse transcription PCR (RT q-PCR) to assess the expression of cytokine genes in Caco-2 monolayers in response to exposure to spirochetes or their products. The Caco-2 cells were grown in 48 well plates and exposed to 95/1000 cells resuspended in DMEM, or other materials, as previously described. In the first experiment the expression of the genes encoding nine cytokines (interferon-γ, tumor necrosis factor-α (TNF-α), interleukin 1β (IL-1β), IL-2, IL-4, IL-5, IL-6, IL-8, and IL-10), as well as β-actin as the internal control, was assessed in triplicate after 2, 4, 8 and 12 h exposure to cultures of 95/1000. In the second experiment only the expression levels of TNF-α, IL-1β, IL-6 and IL-8, and the internal control, were measured. Six replicates of the Caco-2 cells were exposed for 12 h either to DMEM, sterile uninoculated broth, broth supernatant from the 95/1000 culture, a sonicate of 95/1000, 10^8^ cells of 95/1000 in DMEM, or 10^8^ cells of *B. innocens* B256^T^ in DMEM. In both experiments the cells then were rinsed in PBS, trypsinized, and total RNA was isolated from the treated samples using the High Pure RNA Isolation Kit (Roche, Mannheim, Germany), according to the manufacturer's instructions. Complementary DNA (cDNA) was synthesized using the High Capacity cDNA Reverse Transcription Kit (Applied Biosystems, Australia) from 500 ng RNA in a 20 µl reaction. Cytokine mRNA expression was measured using a hot start master mix (FastStart SYBR Green Master; Roche, Mannheim, Germany), according to the manufacturer's instructions.

The primers used for all the cytokines except IL-8 have been described previously [24], whilst the primers specific for IL-8 were designed to target a 136 bp conserved region of the molecule (Forward: 5′-ACCTTTCCACCCCAAATTTATC-3′ and Reverse: 5′-TCTGCACCCAGTTTTCCTTG-3′). The primers for β-actin, a reference gene, have been described previously [25]. Amplification and detection of specific products were conducted on the Rotor-gene 6000 real time PCR system (Corbett Life Science, Mortlake, NSW, Australia), with the following cycle profile: one cycle of 95°C for 10 min, and 40 cycles of 95°C for 20 s and 60°C for 60 s. The expression ratio of each cytokine gene in cells subjected to the specific treatment relative to those incubated with DMEM was calculated using the 2^−ΔΔCT^ method [26]. Within each experiment, fold difference values for the different treatments were compared using analysis of variance (ANOVA), and differences between groups were compared using the Tukey-Kramer Multiple Comparisons test in SPSS Statistics 17.0.

## Results

### Attachment to Caco-2 Monolayers

**Figure 1:**
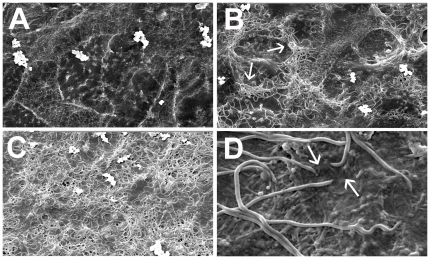
Scanning electron micrographs of *B. pilosicoli* interacting with Caco-2 cells. The cells were incubated for 6 h with DMEM (A), *B. pilosicoli* 95/1000 for 2 h (B), and 6 h (C), and WesB for 6 h (D). The non-infected cells show intact tight junctions with clear boundaries. After 2 h, *B. pilosicoli* 95/1000 mainly colonizes the cell boundaries (arrows), but by 6 h most of the cell surface is covered with spirochetes. The ends of the WesB cells can be seen penetrating the membrane of the Caco-2 cells (arrows), with the rest of the spirochete cell body lying on the Caco2 cell surface. The photographs were taken at magnifications of X 2,100 for panels A, B and C, and X 9,800 for panel D.

The Caco-2 monolayers exposed to DMEM for 6 h remained intact throughout the assays. The cell surface as seen under the scanning electron microscope is shown in Figure 1A, to contrast with subsequent images of attached spirochetes. Following washing and processing, no cells of *B. pilosicoli* strains Karlton or Cof-10 were seen attached to the Caco-2 cells, but strains 95/1000 and WesB were attached. The results of the semi-quantitative attachment scores for the latter two strains are presented in Table 1. For both strains the number of bacteria that were attached increased with time, but the total number was greater for 95/1000 than for WesB. At 2 h, attachment was limited, and mainly confined to the junctions of the Caco-2 cells (Figure 1B). At 4 h, the cell junctions were colonized with large numbers of spirochetes, and more spirochetes were observed overlying the rest of the cell surfaces. At 6 h there was extensive colonization covering all the cell surfaces, especially with strain 95/1000 (Figure 1C). At higher magnifications one cell end of individual spirochetes could be seen indented into the Caco-2 cell membrane, whilst the rest of the body of the spirochete lay over the Caco2 cell surface (Figure 1D).

**Table 1:** Density of attachment of *B. pilosicoli* cells to Caco-2 cells after 2, 4 and 6 h incubation.*

	Strain	
Incubation time (h)	95/1000	WesB
2	2.5 (2–3)	1 (0–2)
4	3 (2–4)	2 (2–3)
6	5 (4–5)	3 (3–4)

* Results are derived from 12 fields of view. They are median (and the range) at each time point, where 0 represents no attachment observed and 5 represents the entire surface of the field covered with spirochetes such that the Caco-2 cell surface was not visible.

**Figure 2:**
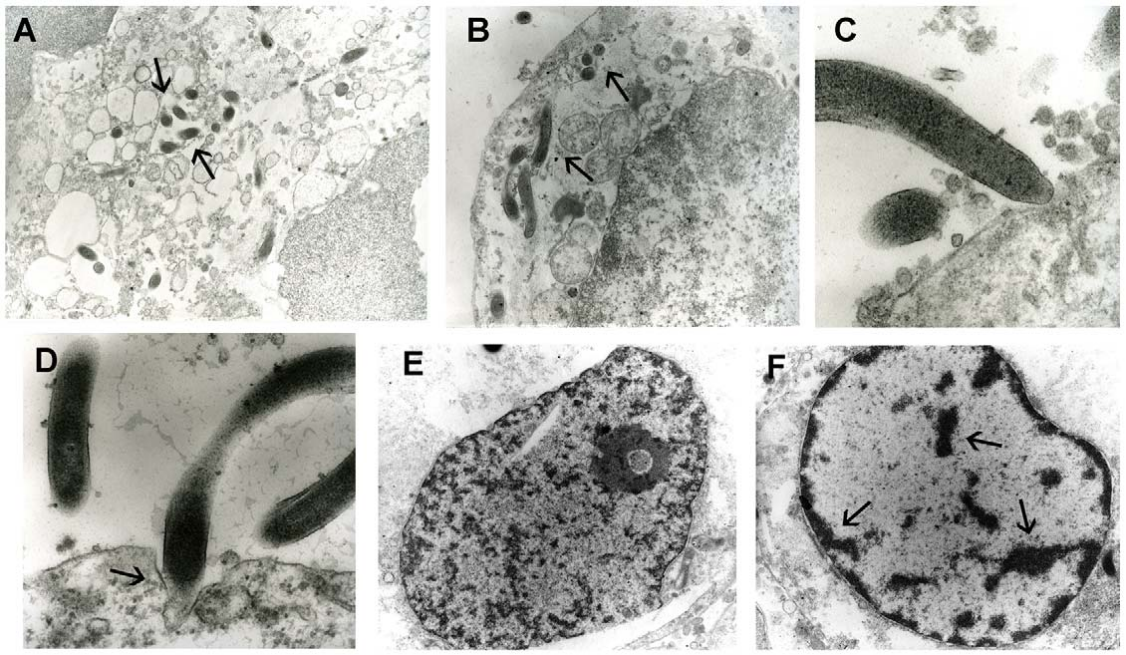
Transmission electron micrographs of *B. pilosicoli* interacting with Caco-2 cells. Cross-sections and tangential-sections of *B. pilosicoli* can be seen at the cell junctions (A) and under the cell membranes (B) (arrows). Spirochete cells can be seen attached to the Caco-2 cell surface (C), and invaginating into pit-like structures (arrow) in the Caco-2 cell membrane (D). Compared to the nuclei of control cells (E), the nuclei of many cells in the infected monolayers show chromatin condensation and fragmentation (arrows), consistent with apoptosis (F). The photographs were taken at magnifications of X 5,800, 7,900, 33,800, 24,500, 5,800 and 5,800, respectively.

Using the TEM, in the infected monolayers tangential-sections and cross-sections of spirochete cells were observed between the cell junctions and associated with the membrane of the Caco-2 cells (Figures 2A and 2B). The TEM further identified end-on attachment of spirochete cells (Figure 2C), and in some cases these were seen invaginating into the membranes of the Caco-2 cells, in a manner similar to the attachment observed *in vivo* (Figure 2D). Compared to the nuclei of cells in the uninfected monolayers (Figure 2E), in the infected monolayers there was a time-dependant increase in the number of cells that had nuclei showing chromatin condensation and fragmentation, consistent with apoptosis (Figure 2F).

### ZO-1 Distribution

**Figure 3:**
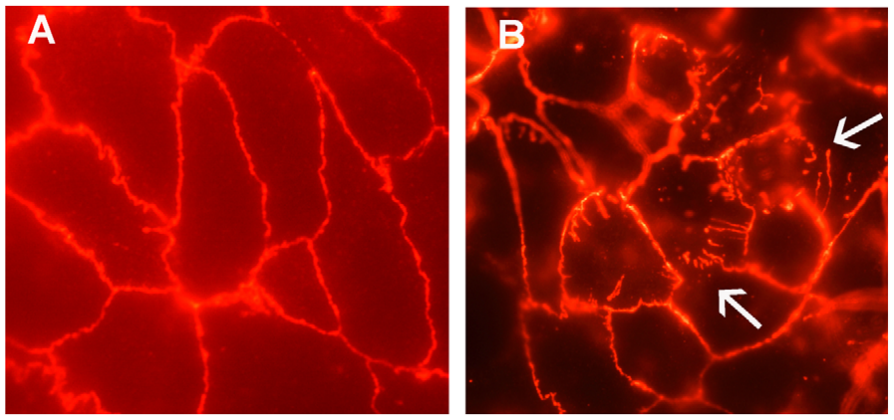
Epifluorescent micrographs illustrating ZO-1 integrity in Caco-2 cell monolayers. Monolayers grown in DMEM (A), and exposed to *B. pilosicoli* 95/1000 for 6 h (B). In the control cells the ZO1 distribution is regular and limited to the junctions, which are intact. After 6 h incubation with *B. pilosicoli* the tight junctions are disrupted and the ZO-1 is punctuated and has migrated towards the cytoplasm (arrow). Photographs taken at a magnification of X 100.

The monolayers exposed to DMEM for 6 h showed intact cell junctions with regular distribution of ZO-1 on the pericellular tight junctions of the Caco-2 monolayers (Figure 3A). After 2 h incubation with *B. pilosicoli* 95/1000, the junctions appeared irregular, were occasionally broken, and some ZO-1 was punctated and had migrated towards the cytoplasm of the cells from the junctions. After 6 h exposure the junctions of many of the Caco-2 cells were disrupted either focally or completely, and the ZO-1 staining was punctated on the junctions. A large amount of ZO-1 had migrated from the junctions towards the centre of the Caco-2 cells, and overall there appeared to be considerable damage to the junctions of the cells (Figure 3B). The supernatant from the *B. pilosicoli* culture did not induce similar changes to the distribution of ZO-1.

### Hoechst Staining of Caco-2 Cell Nuclei

**Figure 4:**
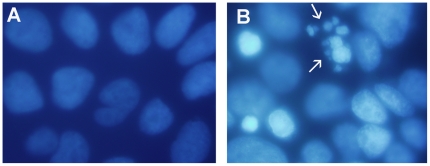
Epifluorescent micrographs showing Hoechst staining of DNA in Caco-2 cells. Monolayers either grown in DMEM (A), or exposed to a culture of *B. pilosicoli* 95/1000 (B) for 6 h. Exposure to *B. pilosicoli* has resulted in many nuclei appearing condensed, and some showing clear chromatin fragmentation, consistent with apoptosis (arrows). Photographs taken at a magnification of X 100.

The control Caco-2 monolayers incubated with DMEM exhibited characteristic uniform fluorescent nuclear staining throughout all nuclei (Figure 4A). A 2 h exposure to the 95/1000 culture induced mild chromosomal condensation and nuclear fragmentation among some cells, with a few nuclei showing intense changes. After 6 h exposure there was considerable condensation and fragmentation of the nucleic acid in many cells (Figure 4B). At this time, the mean and standard deviation of the percentage of nuclei in the six fields showing condensation and/or nuclear fragmentation was 8.8±1.9 in the controls and 35.6±5.8 in the infected cells, and this difference was highly significant (*P*<0.001).

### Actin Rearrangement

**Figure 5:**
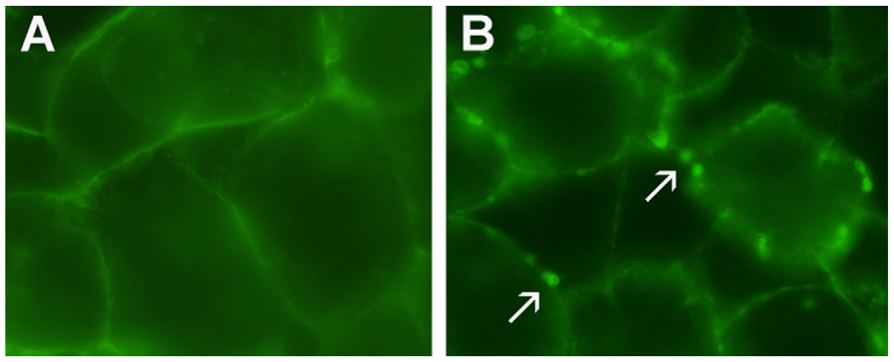
Epifluorescent micrographs showing actin staining in Caco-2 monolayers. Monolayers either grown in DMEM (A), or exposed to *B. pilosicoli* 95/1000 (B) for 6 h. In the control section there is regular distribution of FITC (phalloidin) over the monolayers. After 6 h incubation with *B. pilosicoli* the actin filaments are clearly mobilized and can be seen as round bodies on the junction of the Caco2 cells (arrows). Photographs taken at a magnification of X 100.

In the control cells stained with FITC-phalloidin there was regular distribution of actin filaments at the cell peripheries/junctions (Figure 5A). After 2 h exposure to *B. pilosicoli*, the distribution pattern of actin in the Caco-2 cells was regular, with some actin re-localized in a few places at the periphery of cells. The monolayers exposed for 6 h exhibited an irregular distribution of round or oval concentrations of actin filaments, which was intense at many places on the Caco-2 cell peripheries/junctions (Figure 5B). The culture supernatant did not induce a similar rearrangement of filamentous actin.

### Cytokine Expression

In the initial experiment, the RT q-PCR assays showed that exposure of Caco-2 cells with live *B. pilosicoli* significantly (*P*<0.05) enhanced expression of the IL-1β and IL-8 genes in a time dependent manner, with expression being greatest at 12 h. Expression of TNF-α and IL-6 also increased, but not significantly, while expression of the remaining 5 cytokines was unaltered (data not shown). In the subsequent experiment, examining 4 genes, expression of IL-1β and IL-8 was again significantly up-regulated by incubation with live 95/1000, whilst the sonicate caused a significant up-regulation of TNF-α, IL-1β and IL-6, and a non-significant numerical increase in expression of IL-8 (Table 2). No other treatments caused significant up-regulation of these genes.

**Table 2:** Changes in cytokine expression in Caco-2 cell monolayers.*

	Materials incubated with the Caco-2 cells						
Cytokine	DMEM	Sterile broth	Culture supernatant	Culture sonicate	Live B. *pilosicoli*	Live B. *innocens*	P value in ANOVA
IL-1β	1.05^a^±0.36	1.11^a^±0.52	1.18^a^±0.57	**21.25^b^±12.97**	**17.72^b^±4.12**	2.24^a^±2.71	<0.0001
TNF-α	1.06^a^±0.36	1.02^a^±0.25	1.42^a^±0.49	**24.92^b^±5.84**	5.51^a^±3.46	4.61^a^±6.84	<0.0001
IL-6	1.32^a^±0.71	1.26^a^±0.72	5.68^a^±3.07	**37.12^b^±16.60**	1.04^a^±0.81	2.83^a^±3.65	0.0005
IL-8	1.05^a^±0.35	1.19^a^±0.67	1.83^a^±0.79	6.75^a^±5.96	**25.33^b^±19.05**	1.62^a^±2.12	0.0003

* Monolayers exposed for 12 h to DMEM, sterile broth, *B. pilosicoli* strain 95/1000 culture supernatant and sonicates, live *B. pilosicoli* 95/1000, and live non-pathogenic *B. innocens* B256^T^. The results are mean ± standard deviation of fold difference in gene expression measured by RT-qPCR. For each cytokine, treatment means marked with a different superscript differ at least at *P*<0.05, and the significantly elevated values are marked in bold.

## Discussion

The ability of bacteria to adhere is one of the essential features required for successful colonization of the gastrointestinal tract [27]. In both natural and experimental infections with *B. pilosicoli* in humans, animals and birds, the spirochete shows an unusual form of attachment to the surface of colonic enterocytes, whereby one cell end pushes against and then invaginates into the cell surface, without penetrating the cell membrane [3], [28]. Subsequently, specific interactions with the host cell appear to occur, anchoring the spirochete in place within the pit-like structure that is formed [29]. In the current study, a similar form of attachment to Caco-2 cells was obtained (Figures 1D and 2D). Although with time the spirochetes came to overlay and blanket the whole surface of the cells, closer examination showed that many of the individual cells were anchored to the Caco-2 cells by one end. The spirochetes did not form a “false brusg border” with a palisade of upright cells perpendicular to the Caco-2 cell surface, but this may only reflect the lack of a thick mucus blanket over the Caco-2 cells which is likely to support this orientation *in vivo*. The high multiplicity of infection that was used also may have contributed to the way the spirochetes were layered over the Caco-2 cells, whereas in natural infections the palisades may develop gradually as individual attached spirochetes divide and the new cell also attaches.

In this study only porcine strain 95/1000 and human strain Wes B attached to the Caco-2 cells. The other two strains were completely removed following the washing and fixing process. The basis for there being differences in attachment with different *B. pilosicoli* strains is not known. All four strains were actively motile, were at a similar passage number, and were used at the same multiplicity of infection. Differences amongst the strains in their specific surface proteins and/or glycans that may interact with receptors on the Caco-2 cell surface could help to account for their different ability to attach. Strains of *B. pilosicoli* also have been shown to vary in their attraction to mucin *in vitro*, and it was of interest that strain Cof-10 that did not attach in the current study also was not attracted to mucin [30]. It will be important to determine whether these *in vitro* activities of the different strains reflect how they behave *in vivo*, and whether the *in vitro* data can be used to help predict the virulence potential of a given isolate.

The initial site of attachment on the monolayer was mainly around the cell junctions, and then, with time, attached spirochetes were observed over the rest of the cell surface. A similar pattern of attachment at the cell junction has been seen *in vivo*, and it has been suggested that this distribution may facilitate penetration of the epithelial layer [28]. This intercellular route may be the way by which *B. pilosicoli* is able to translocate through the colonic epithelium, and enter the bloodstream [31]. The reasons for the subsequent spread of attachment from the junctions over the whole cell surface are not obvious, but it is possible that spirochete-induced changes occurred in the Caco-2 cells that made them more receptive to the spirochetes.

Analysis of TEM micrographs and Hoechst staining indicated that in the cells exposed to *B. pilosicoli* 95/1000 there was a time-dependant condensation and fragmentation of the nuclear material, consistent with apoptosis. In future work, pre-treatment with specific inhibitors, such as a caspase-3 inhibitor, could be used to help to confirm the occurrence of apoptosis [32]. Similarly, using ZO-1 staining, a time-dependant disruption to the zonula occludens was observed. It was unclear whether subsequent changes in cellular permeability may have initiated apoptosis, or *vice versa* [32], [33]. The disruptions at the cell junctions were not induced by the culture supernatant, and hence toxic products released into the medium from the growing spirochetes were not responsible for the damage. Currently little is known about how *B. pilosicoli* could induce localized damage, although the spirochete is known to possess membrane-associated serine proteases and other proteases [34]. It is possible that the spirochete produces and delivers other toxic molecules directly at the cell surface. Some examples of such bacterial toxins that act at the cell junction include the fragilysin toxin produced by *Bacteroides fragilis*, causing the degradation of the ZO-1 protein [35], and the *Clostridium difficile* toxins TcdA and TcdB that cause the dissociation of occludin, ZO-1, and ZO-2 [36].

Specific staining revealed a time-dependant accumulation of filamentous actin at the cell margins, and this is the first report providing evidence for actin rearrangement associated with *B. pilosicoli* colonization. An accumulation of actin was not previously seen in monolayers where the spirochetes showed only a diffuse non-polar attachment [22], and this observation supports the likely existence of a causal association between the polar attachment and these specific changes. The ability of certain bacteria to manipulate the host's cytoskeleton in such a way is known to be important for adhesion and invasion [37]–[39], and further work is required to confirm this observation and elucidate the specific mechanisms involved in this interaction. Earlier work using gene probes suggests that the mechanisms involved are likely to be different to those that occur with enteropathogenic *E. coli*, *Y. enterocolitica* or *S. flexneri* [15]. Further insight into possible effectors and mechanisms may become available once the full genome sequence of *B. pilosicoli* becomes available.

In order to help identify the responsiveness of the Caco-2 cells to the attachment by *B. pilosicoli*, assays were undertaken to assess the expression of selected cytokines by the monolayers. In a pilot experiment, exposure to live attaching 95/1000 cells induced a significant time-dependent increase in expression of the genes encoding IL-1β and IL-8, and some increases in TNF-α, and IL-6. The increase in IL-1β and IL-8 expression was confirmed in the second experiment, where a sonicate of 95/1000 also was shown to induce significant increases in expression of IL-1β, TNF-α, and IL-6. The observation that *B. pilosicoli* culture supernatants caused no change in cytokine mRNA expression is informative, since it suggests that cell-free *B. pilosicoli* toxins or by-products were not involved in the stimulatory effects. IL-1β is an important mediator in intestinal inflammation, promoting production of the pro-inflammatory chemokine IL-8, so it was interesting that both live cells and sonicate of *B. pilosicoli* stimulated its expression. On the other hand, only live *B. pilosicoli* induced significant expression of IL-8, suggesting that induction of this gene may be involved in the process of generating the focal tissue damage and colitis that can occur *in vivo*. Taken together, these findings support the likelihood that *B. pilosicoli* has pathogenic potential. Many other enteric bacterial pathogens similarly induce IL-8 production in cultured enterocytes [40], with, for example, both bacterial motility and adherence being important for this induction in the case of *Vibrio cholerae* [41], and adherence and probably the presence of lipopolysaccharide in the case of *Helicobacter pullorum* [42]. Currently it is unclear what *B. pilosicoli* mediators and Caco-2 cell surface receptors, transduction pathways and transcription factors are involved in generating the up-regulation, although a range of different bacterial products and corresponding Toll-like or other surface receptors on the Caco-2 cells could be involved. This could be investigated further using purified *B. pilosicoli* cell-surface components, together with antagonists of specific surface receptors or intracellular signaling cascades. Again it was interesting that the sonicate caused significant up-regulation of the pro-inflammatory cytokines IL-1β, TNF-α, and IL-6, presumably in response to liberated materials present in the cellular debris. The fact that IL-8 was not significantly upregulated by the sonicate suggests that there may be a specificity in the spirochete attachment process that is involved in generating IL-8 expression.

### Implications

The study has demonstrated that strains of *B. pilosicoli* vary in their ability to attach to Caco-2 cells, and as such they may vary in their ability to colonize *in vivo*. Caco-2 cells that are exposed to attaching strains of *B. pilosicoli* undergo a series of changes, including accumulation of actin at the cell junctions, disruption to the cell membrane, apoptosis, and up-regulation of IL-1β and IL-8. Taken together, these results add to the available evidence demonstrating that strains of *B. pilosicoli* can induce pathological changes, and provide a basis for explaining the focal colitis that may be seen in birds, animals and humans who are colonized with *B. pilosicoli*. In future work, more extensive transcriptomics analysis and the use of specific antagonists may help to identify some of the pathways and processes involved in the interactions.
